# Commonality Between Diabetes and Alzheimer’s Disease and A New Strategy for the Therapy

**DOI:** 10.4137/cpath.s667

**Published:** 2008-07-28

**Authors:** Li Lin

**Affiliations:** Key laboratory of cellular physiology, Shanxi Medical University, China

**Keywords:** glucagon-like peptide 1, diabetes, Alzheimer’s disease

A surprising common pathological processes is found between Alzheimer’s disease (AD) and type 2 diabetes mellitus (T2DM)^[1]^. AD and T2DM share some common pathological processes: Amyloid β (Aβ), τ hyperphosphoralation, insulin abnormality. Disturbance in insulin signalling is not only involved in blood glucose level but also in numerous degenerative processes. Glucagon-like peptide 1 (GLP-1) has attracted substantial attention for its advantage in treating T2DM. GLP-1 can reduce Aβ levels in brain. All these encourage us in a medical hypothesis: that is, that GLP-1 is a promising agent in the therapy of AD.

## Commonalities Between T2DM and AD

Both AD and T2DM are the most common degenerative diseases and their prevalence increase with age. Numerous epidemiological studies have linked T2DM with an increased risk of AD^[2]^. An investigation of cohort of 1,301 in Stockholm, Sweden showed that T2DM increased the risk of dementia^[3]^. In another survey of 683 subjects cohort of persons aged 65 years and older with hyperinsulinemia in northern Manhattan is shown to be associated with a higher risk of AD and decline in memory^[4]^. In a prospective population-based cohort study among 6,370 elderly subjects, Ott^[5]^ revealed that diabetes may have contributed to the clinical syndrome in a substantial proportion of all dementia patients. Peila^[6]^ evaluated the association of diabetes alone or combined with the apolipoprotein E (ApoE) gene with incident dementia in a population-based cohort of 2,574 Japanese-American men. The result displayed that T2DM is a risk factor for AD. The association between diabetes and AD is particularly strong among carriers of the APOE epsilon4 allele.

Commonalities between T2DM and AD in epidemiology: such as degenerative change, aging diseases, fat and higher cholesterol, and cardiac risk factors, encourage us to look for the linkage of both. Research has shown an extensive expression of insulin and its receptor throughout the mammalian brain^[7,8]^. A fact of commonalities between neurons and β-cells launch into a reasonable explanation as to the common risk and prevention factors between T2DM and AD. Research has shown some common risk factors between T2DM and AD: higher cholesterol^[9]^, dis-metabolism, degeneration^[9]^, myloid β (Aβ) deposition^[9]^, Glycogen synthesis kinase 3(GSK3), and τ protein phosphorylation^[10]^, cardiovascular disease, oxidative stress^[2]^, inflammation^[2]^, ApoE4^[11]^, apoptosis etc. A new finding shows that treatment with GLP-1 beneficially affects a number of the therapeutic targets associated with AD. This finding opens a new research area. The commonalities between T2DM and AD may contribute to the explanation of GLP-1 as a promising peptide to treat T2DM and AD.

AD is characterized by intracellular neurofibrillary tangles (NFTs), containing an abnormal hyperphosphorylated form of τ protein, and extracellular senile plaques (SPs), mainly composed of fibrillar Aβ. Both of those hallmarks are involved in T2DM^[1]^. Both age-related degenerative diseases, AD and T2DM, are associated with the accumulation of amyloid fibrils^[12]^. Another commonality, cell loss and degenerative change, is also involved in both diseases. AD is the most common neurodegenerative disease with an extensive neuron loss. T2DM is also a degenerative disease that results from the selective destruction of pancreatic β cells^[13]^.

Aβ deposits are commonly observed in pancreatic islets of diabetic patients. These deposits consist of islet amyloid polypeptide (IAPP)^[14]^.Considering the pathogenetic similarities and the 90% structural similarity between Aβ precursor protein and IAPP^[15]^, it should not be surprising that AD seems to be predisposed to insulin resistance, insulin hypersecretion, and T2DM^[2]^. Similarly, individuals suffering from T2DM will suffer from dementia more readily^[5,16]^. The research has shown that a higher serum insulin level in prediabetes and early T2DM has been associated with impaired cognitive function^[17]^. Mechanistically this might be that elevation of Aβ levels is associated with elevated serum insulin content^[18]^. In other words, the evidence of the existence of links between AD and T2DM is that AD is associated with peripheral and central insulin abnormalities, and that cognitive capacities are often impaired in patients with T2DM^[14]^. The complex relationship between insulin, cholesterol, and AD was well described by Nelson^[19]^. Insulin regulates cholesterol biosynthesis by stimulating activity of 3-hydroxy-3-methylglutaryl-CoA reductase, a rate-limiting enzyme in cholesterol biosynthesis. Cholesterol is involved in AD by multifactor: ApoE4, Aβ deposition, amyloid precursor protein (APP) metabolism etc. Hypercholesterolemia is an obvious risk factor of T2DM^[20]^. It was shown that transgenic mice models overexpressing IAPP develop diabetes and generally subsequent to amyloid deposits^[21,22]^. Conversely, targeted disruption of IAPP leads to enhanced insulin secretion and improved glucose tolerance^[23]^.

Freude^[24]^ showed a correlation between τ phosphorylation and peripheral insulin level with a significant increase in τ phosphorylation at Ser^202^ in the brain within 10 min after 1-mU insulin injection and an even further increase after injection of 4 units insulin. Further, Freude demonstrated that insulin receptor signaling and τ phosphorylation were completely abolished in the brains of the mice lacking the brain insulin receptor under hyperinsulinemic conditions, indicating that the cerebral insulin receptors are a direct target of peripheral administered insulin. Further evidence of τ phosphorylation involvement in AD and T2DM is that GSK-3, a serine/threonine kinase that phosphorylates glycogen synthase in the rate-limiting step of glycogen biosynthesis, was implicated in the formation of NFTs^[25,26]^. GSK-3 inhibitor is an attractive target identified to be useful in the treatment of diseases such as T2DM and AD^[27]^.

Common biological characteristics between the pancreas and the brain also include common enzymes: glutamic acid decarboxylase, tyrosine hydroxylase, and dopa decarboxylase, thyrotrophin-releasing hormone, and P75. Neuron growth factor receptors are also shared attributes of both neuronal tissues and β-cells. β-cells resemble neurons in that they are electrically excitable and respond to hormonal stimuli and glucose by depolarization and exocytosis, in a process that resembles neurotransmitter release from synaptic vesicles. All those commonalities approve a reasonable assumption that common signaling mechanisms occur in response to similar physiological responses, such as proliferation and differentiation^[28]^. Furthermore the impact of type 1 diabetes on brain development and function has been reviewed by Northam^[29]^. Either hypoinsulinemia or hyperinsulinemia are involved in the damage of brain development.

There is growing evidence that insulin is involved in cognitive decline and AD^[30]^. The evidence includes the insulin resistance of brain owing to alterations of insulin receptor signaling in the brain, and regulation of insulin to the metabolism of Aβ and τ protein. It is further evidence that there is widely distribution of insulin and insulin receptor (IR) in the brain, especially in the hypothalamus and the hippocampus^[31]^. The hippocampus- and cerebral cortex-distributed insulin/IR has also been shown to be involved in brain cognitive functions. In contrast, deterioration of insulin receptor signaling is involved in aging-related brain degeneration such as the AD and cognitive impairment in T2DM patients^[31]^. Moroo^[32]^ showed a decrease of expression of IR in the brain in AD and PD patients. Frolich^[33]^ compared the expression of insulin and its IR in neocortical brain areas of AD patients with normal controls by immunohistochemical staining. The result showed that insulin and its receptor densities decrease with aging. Brain IR densities in AD were decreased compared to middle-aged controls. The effect of insulin in AD patients includes: 1) AD may be associated with an impairment of glucose regulation. 2) AD may worsen insulin abnormalities. 3) AD patients may have a decreased cerebrospinal fluid insulin levels and/or a decreased cerebrospinal fluid-to-plasma insulin ratios. 4) Acute glucose administration can facilitate memory of AD patients and healthy older adults; however, it is abolished by suppressing endogenous insulin secretion. 5) Acute insulin administration facilitates memory of AD patients. 6) Apo E does not only involve in AD but also moderates insulin activity and affects on memory of patients with AD. Patients without an APOE ɛ4 allele have lower insulin sensitivity and occur insulin-induced memory facilitation at higher insulin doses. Reversely, Patients with at least one APOE q4 allele show insulin-induced memory facilitation at lower insulin doses and reduced insulin degrading enzyme levels. Hence, disturbances in cerebral insulin signalling pathways may be involved in AD and brain aging^[34]^.

The relation between insulin and the metabolism of Aβ and τ is also receiving increasing attention. Insulin appears to stimulate Aβ secretion and inhibits the extracellular degradation of Aβ due to competition from insulin-degrading enzyme (IDE)^[35]^.

Hyperglycaemia may be involved in the brain damage. Hyperglycaemic rodents, for example, express cognitive impairments and functional and structural alterations in the brain^[36]^. Schubert^[37]^ hypothesized that neuronal insulin resistance contributes to defects in neuronal function and exhibited the evidence in insulin receptor knockout (NIRKO) mice. A complete loss of insulin-mediated activation of phosphatidylinositol 3-kinase and inhibition of neuronal apoptosis was shown in NIRKO mice. As a result, phosphorylation of GSK3 was markedly reduced and phosphorylation of τ protein increased. The hypothesis should be further completed because Schubert has not exhibited the alteration of neuronal proliferation survival, memory, or basal brain glucose metabolism in NIRKO mice. Involvement of other factor will be needed to develop AD based on changes in GSK3 activity and hyperphosphorylation of τ protein induced by lack of insulin signaling in the brain.

**Figure 1.**
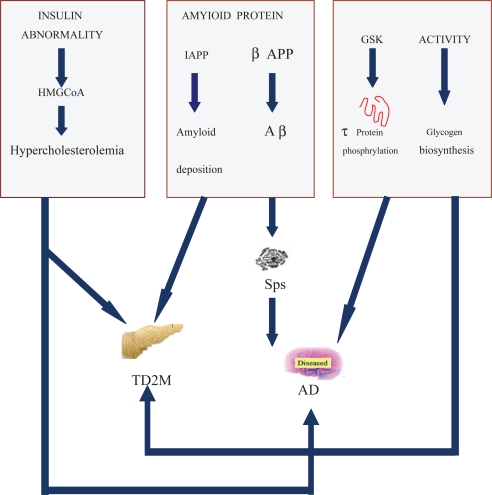
Common pathological processes in AD and T2DM. Ninety percent homologous structure was shown between Aβ which is a hallmark pathology in AD and IAPP which is involved in T2DM. Insulin abnormality is attributed to AD by promoting Aβ deposite, and τ protein hyperphosphorylation. GSK3 is a key kinase to promoteτ protein hyperphosphorylation and glycogen biosynthesis. **Abbreviations:** AD: Alzheimer’s disease; T2DM: Type 2 diabetes mellitus; IAPP: islet amyloid polypeptide; Aβ: Amyloid β; GSK3: glycogen synthase kinase.

A complex relationship between diabetes and AD is shown in the Figure 1. Ninety percent structural similarity was founded in Aβ which is a hallmark pathology in AD and IAPP which is involved in T2DM. Insulin abnormality is attributed to AD by promoting Aβ deposition, and τ protein hyperphosphorylation. GSK3 is a key kinase to promote τ protein hyperphosphorylation and glycogen biosynthesis.

## A New Strategy Consideration to Treat AD

A cure of AD is still far off, and clinicians face the burden of caring for patients at all stages of dementia for the foreseeable future^[38]^. Heretofore, therapy strategies of AD, including the cholinesterase inhibitors, are only limited in heteropathy: improving cognitive impairment, decreasing complication, preventing abnormity behaviors, and avoiding psychopathic affair. Effective strategies aimed at the hallmarks of AD, and developing therapies that target Aβ production, aggregation, clearance or toxicity are likely to appear in the near future^[39,40]^. The therapy strategy aimed at other hallmarks of AD, neurofibrillary tangles, is also being developed^[41]^.

Even with treatment, patients with T2DM may face some troubles: spikes in blood glucose after meals, weight gain, a loss of effectiveness of their treatments over time etc. GLP-1 draws on a better understanding of how the body responds to meals—some already available and may offer useful adjuncts to existing therapies^[42]^.

Emerging literature, in which it is shown that hallmarks of AD can link to T2DM, and GLP-1 can reduce Aβ-peptide levels in the brain, encourage an adventurous thinking: is GLP-1 a new hope for the therapy of AD?

## Synthesis, Secretion and Function of GLP-1

GLP-1 originates from expression of the glucagon gene in the L cells of the distal intestinal mucosa and contains 30 amino acids with 50% sequence homology to glucagons^[43–45]^. GLP-1 was found as an insulinogenic factor 20 years ago. In all candidate incretins, insulinogenic factors of the gastrointestinal mucosa, GLP-1 was regarded as the most physiological signification, possessing obvious characters of gastrone and insulinotropic substances^[46]^. Its effects on glucose-dependent insulin secretion and insulin gene expression have been proven^[47,48]^. The mechanism of GLP-1 to regulate blood glucose include 1) the stimulation of insulin secretion and of its gene expression, 2) the inhibition of glucagon secretion, 3) the inhibition of food intake, 4) the proliferation and differentiation of β cells, and 5) the protection of β cells from apoptosis^[49]^. 6) formation of pancreatic islet mass.

New discovery that GLP-1 and Exendin-4 (Ex-4), a naturally occurring stable analogue of GLP-1 that binds at the GLP-1 receptor (GLP-1R), possess neurotrophic properties and can protect neurons against glutamate-induced apoptosis and reduce level of Aβ in brain, prompt a new consideration: GLP-1 and its mimics are promising agents in therapy of AD. GLP-1 is also regarded as a promoting agent not only for T2DM but also for AD due to its extensive expression of GLP-1R in the brain^[50–52]^.

In the human genome, the proglucagon gene is located on chromosome 17 spanning approximately 10 kb. The transcriptional unit of proglucagon contain six exons and five introns^[53]^. The posttranslational processing of preproglucagon differs in different tissue. They are, in the pancreas, GLP-1 (1–36 amide) and GLP-1 (1–37), while, in the ileum and hypothalamus, are GLP-1 (7–36)-amide and GLP-1 (7–37)^[54]^. In the pancreatic islet alpha-cells proglucagon is processed by proprotein convertase 2 to release mainly glucagon. In the intestinal L cells it is by proprotein convertase to produce mainly GLP-1, GLP2^[55]^. Amidation of GLP-1 has been regard as a chemical process to enhance its survival in the bloodstream. In the pancreas, the carboxyl-terminal amidation of GLP-1 is processed by the sequential enzymatic action by peptidylglycine α-monooxygenase and peptidy-lamidoglycolate lyase^[56]^. The significance of the amidated forms of GLP-1 in nonpancreatic targets is not clear. GLP-1 has a very short half-life (1–2 min) and is rapidly degraded in vivo by dipeptidyl peptidase-IV (DPP-IV), which cleaves GLP-1 at the penultimate N-terminal site of Ala^8[57–59]^. Fortunately, all GLP-1 analogues have been developed to possess a longer half-life. In vivo, several metabolites of GLP-1 are formed by enzyme digestion. They are GLP-1 (9–36), GLP-1 (7–35), and GLP-1 (7–34). GLP-1 (9–36) amide, a main metabolite of GLP-1, is present in vivo in concentrations that are up to 10-fold greater than the level of GLP-1(7–36) amide^[60]^. Isoforms of GLP-1 show different bioactivity; the effect of GLP-1 (7–36) amide is 100 times more potent than GLP-1 (1–37) and GLP-1 (1–36) amide in stimulating [^14^C]-aminopyrine accumulation^[61]^; GLP-1 [7–36 amide] and GLP-1 [7–37] possess an efficiency^[62]^.GLP-1 (9–36) amide has been shown to have no effects on β cells and it is in some studies shown as an antagonist of the adenylyl cyclase activity^[63]^. The research also showed that GLP-1 (7–35) and GLP-1 (7–34) are an agonists. GLP-1 (7–36) and GLP-1 (7–37) are two of the main naturally occurring GLP products in vivo and the similar insulinotropic potency is proven. The structural analysis of GLP-1 shows that the first seven amino acid residues form a random coil structure followed by a first helical region (7–14), a linker region (15–17) and another helical region (18–29). Plasma levels of GLP-1 rise rapidly following nutrient ingestion. Major regulating factors of GLP-1 secretion are pancreatic hormones (insulin and glucagon)^[64,65]^, nutrients (glucose and fatty acids), gastrointestinal hormones (gastric inhibitory polypeptide), gastrin-releasing polypeptide, gastric emptying^[66,67]^, satiety^[68]^, body weight^[69]^, the vagal nerve-dependent release of acetylcholine etc^[70,71]^. Glucose-dependent Insulinotropic Peptide was also shown as an attractive stimulator of GLP-1 release in vitro^[72]^. GLP-1 secreted from intestinal L cells is inactivated by DPP-IV in intestinal capillaries. DPP-IV deactivates GLP-1 by cleavage of the N-terminal dipeptide. Thus only 25% of GLP-1 secreted enters the portal circulation in its intact form. In the liver 40% of the remaining active GLP-1 is further inactivated. As a result, only 10%–15% of GLP-1 reaches the systemic circulation and the pancreas. GLP-1 imposes on the brain by activating sensory efferent neurons from the nodose ganglion, the hepatoportal region^[73,74]^ or the liver^[75]^, and stimulating the neural pathway^[76]^.

GLP-1R, a member of the seven-membrane-spanning G-protein-coupled family of receptor, is localized on chromosome 6 with 12 introns and 13 exons^[49,77]^. GLP-1R have been identified in brain, lung, pancreatic islets, stomach, hypothalamus, heart, intestine, and kidney^[78,79]^. GLP-1R consists of 463 amino acids (with a molecular weight of 65000 D) with eight hydrophobic domains. The hydrophobic segment located N-terminal is probably a signal area, whereas the others are membrane-spannin hydrophobic motifs^[80]^. GLP-1 receptor efficiently stimulate insulin secretion by coupling with adenyl cyclase^[81]^.

## GLP-1, an Attractive Agent to Treat T2DM and AD

Numerous researches has shown that GLP-1 is an attractive agent in therapy of T2DM^[42,59,82,83]^. Its pharmacological actions in glucose metabolism, including stimulation of insulin release, suppression of glucagon release, and inhibition of gastric emptying, ensure the rationale for its assessment as a therapeutic agent for T2DM^[84]^. The research recently focusing on intervening AD showed that GLP-1 is also a strong intervenor of AD^[28,51,52]^.

GLP-1 regulates insulin secretion and insulin gene expression via its action on the pancreas following binding at the G-protein coupled GLP-1R. The GLP-1R signaling has been demonstrated to inhibit glucagon secretion^[85]^. It has been demonstrated that the commonality of expression of GLP-1R in both the rat and human brain exists^[86]^. Within the central nervous system (CNS), GLP-1 and several analogs that bind it to the GLP-1R possess neurotrophic properties and offer protection against glutamate-induced apoptosis and oxidative injury in cultured neuronal cells^[87]^. Moreover, GLP-1 can modify the processing of Aβ precursor protein in cell cultures and reduce Aβ levels in the brain in vivo^[88]^.

The stimulus for neuronal GLP-1-transmission within the CNS is unclear. One possibility is that GLP-1-containing neurons or receptors are implicated in other neuropeptide-containing CNS pathways or down-stream from classic neurotransmitter systems such as noradrenalin, serotonin or dopamine. An alternative possibility is that peripheral GLP-1 acts on vagal afferent fibers^[89]^, where it could influence GLP-1 neuronal transmission in the CNS.

GLP-1R positively regulates neuronal plasticity and cell survival as the stimulation of neuron^[51]^. It was recently reported that GLP-1 and Ex-4 possess neurotrophic properties and can protect neurons against glutamate-induced apoptosis. Perry^[87]^ showed that GLP-1 can reduce the levels of Aβ in the brain in vivo and can reduce levels of APP in cultured neuronal cells. GLP-1 and Ex-4 protect cultured hippocampal neurons against death induced by Aβ and iron, an oxidative insult. Collectively, these data suggests that GLP-1 can modify APP processing and protect against oxidative injury. During^[90]^ showed that intracerebroventricular exendin enhanced associative and spatial learning and prevented kainate-induced apoptosis of hippocampal neurons in rats. In recent research, Perry^[91]^ showed that GLP-1 (Ex-4) has multiple synergistic effects on glucose-dependent insulin secretion pathways of pancreatic β-cells and on neural plasticity. Their study showed that GLP-1 (Ex-4) may offer some protection against the sensory peripheral neuropathy induced by pyridoxine. Put those data together it seems a potential role for these peptides to treat neuropathies.

Parsons^[92]^ has made a GLP-1 minigene that can direct the secretion of active GLP-1 (amino acids 7–37) to achieve continuous GLP-1 expression to lengthen its half-life in vivo. In order to delay half-life and improve the therapeutic value of GLP-1, Youn^[93]^ chemically modified GLP-1 with polyethylene glycol (PEG) and proved that the site-specific Lys^34^-PEG-GLP-1 was found to have significantly improved in vivo glucose-stabilizing efficacy than the other PEGylated GLP-1 isomers (His^7^- or Lys^26^-PEG-GLP1). Nevertheless, some of the GLP-1-derived agonists with DPP-IV resistance appear to be rapidly cleared from the plasma by renal clearance. The clinical studies of Ex-4 showed that daily administration or combination therapy with oral anti-diabetic agents was required to normalize blood glucose levels. To develop the longer-acting molecules that retain the native GLP-1 actions is a motivated effort. It is also clear that a gene therapy approach exerting long lasting effects would have advantages over parenteral protein drug delivery. A new gene therapy agent: GLP-1/IgG1-Fc fusion construct developed by Kuma^[94]^ showed a unique advantage. Fusing active human GLP-1 and mouse IgG_1_ heavy chain constant regions (GLP-1/Fc) Kuma generated a plasmid encoding an IgK leader peptide-driven secretable fusion protein of the active GLP-1 and IgG_1_-Fc was constructed for mammalian expression. The researches in vivo and vitro showed that the bivalent GLP-1/Fc fusion protein is an effective approach for the therapy of T2DM.

In clinic treatment, GLP-1 based therapies for T2DM has made progress. Therapy strategies related to GLP-1 include injected DPPIV-resistant GLP-1 mimetics or orally active DPPIV inhibitors^[75,95,96]^. The GLP-1 enhancers and DPPIV inhibitors in pre-registered by FDA are Vildagliptin (LAF237), Sitagliptin (MK-0431); in clinical phase III programs are Denagliptin, SYR 322, Saxagliptin (BMS477118); in clinical phase II are Ro-0730699, PSN 9301, TA 6666. GLP-1 mimetic, Byetta-Exenatide, has been launched in U.S. (2005). GLP-1 minetics in clinical phase III programs is Liraglutide NN2211, in clinical phase II programs are ZP-10, BIM-51077, Exenatide-LAR, and CJC-113^[97]^.

In summary, with a century of studies, the understanding of AD leads us to believe that the primary targets in AD are the Aβ and τ protein. Commonalities between T2DM and AD and the effect of GLP-1 on Aβ encourages us to forecast the possibility that GLP-1 is revealed as the highlight in further T2DM treatment and will bring forth its advantage in the therapy of AD.
